# Author Correction: Lipid nanoparticle encapsulated nucleoside-modified mRNA vaccines elicit polyfunctional HIV-1 antibodies comparable to proteins in nonhuman primates

**DOI:** 10.1038/s41541-021-00397-2

**Published:** 2021-11-06

**Authors:** Kevin O. Saunders, Norbert Pardi, Robert Parks, Sampa Santra, Zekun Mu, Laura Sutherland, Richard Scearce, Maggie Barr, Amanda Eaton, Giovanna Hernandez, Derrick Goodman, Michael J. Hogan, Istvan Tombacz, David N. Gordon, R. Wes Rountree, Yunfei Wang, Mark G. Lewis, Theodore C. Pierson, Chris Barbosa, Ying Tam, Gary R. Matyas, Mangala Rao, Zoltan Beck, Xiaoying Shen, Guido Ferrari, Georgia D. Tomaras, David C. Montefiori, Drew Weissman, Barton F. Haynes

**Affiliations:** 1 Duke Human Vaccine Institute, Duke School of Medicine, Durham, NC USA; 2 Department of Surgery, Duke School of Medicine, Durham, NC USA; 3 Department of Molecular Genetics and Microbiology, Duke School of Medicine, Durham, NC USA; 4 Department of Immunology, Duke School of Medicine, Durham, NC USA; 5 Department of Medicine, University of Pennsylvania, Philadelphia, PA USA; 6 Department of Medicine, Duke School of Medicine, Durham, NC USA; 7 Beth Israel Deaconess Medical Center, Boston, MA USA; 8 Bioqual Inc., Rockville, Maryland, USA; 9 Laboratory of Viral Diseases, National Institute of Allergy and Infectious Disease, National Institutes of Health, Bethesda, MD USA; 10 Acuitas Therapeutics, Vancouver, BC, CA USA; 11 Laboratory of Adjuvant & Antigen Research, US Military HIV Research Program, Walter Reed Army Institute of Research, Silver Spring, MD 20910 USA; 12 Henry M. Jackson Foundation for the Advancement of Military Medicine, Bethesda, MD 20817 USA; 13 Present Address: Pfizer, 401 N Middletown Rd, Pearl River, NY, 10965 USA

**Keywords:** Vaccines, RNA vaccines, Antibodies, Protein vaccines

Correction to: *npj Vaccines* 10.1038/s41541-021-00307-6, published online 09 April 2021

A number of authors were mistakenly omitted from the original version of this Article. The HTML and PDF versions of the Article have now been corrected.

