# Supplementary material for: Lipid nanoparticle encapsulated nucleoside-modified mRNA vaccines elicit polyfunctional HIV-1 antibodies comparable to proteins in nonhuman primates
Source: NPJ Vaccines. 2021 Apr 9;6:50. doi: 10.1038/s41541-021-00307-6 (PMC8035178; doi:10.1038/s41541-021-00307-6)
Supplement: Supplementary file 1 — Supplemental Information [file 41541_2021_307_MOESM1_ESM.pdf]

Supplementary Figure 1

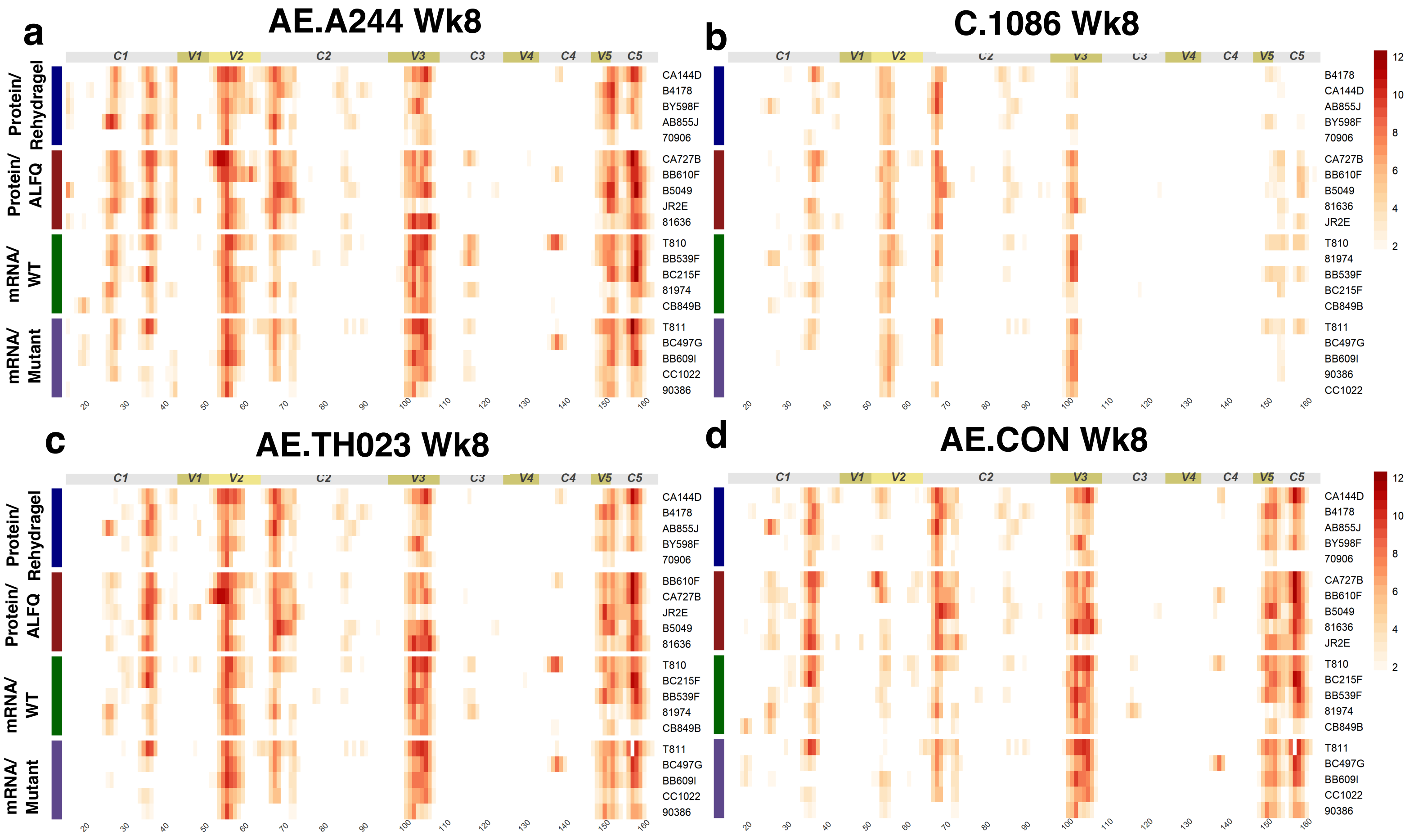

Supplementary Figure 1. mRNA-LNP and adjuvanted recombinant protein vaccination elicits plasma IgG with cross clade specificities. a-d. Heatmap of week 8 plasma IgG binding to peptides spanning the gp120 of **a.** AE.A244, **b.** C.1086, **c.** AE.TH023, and **d.** AE.CON. Each row shows the binding for an individual macaque. The macaque names are shown at the right side of the graph. Segments of HIV-1 gp120 are indicated across the top of the heatmap.

Supplementary Figure 2

a

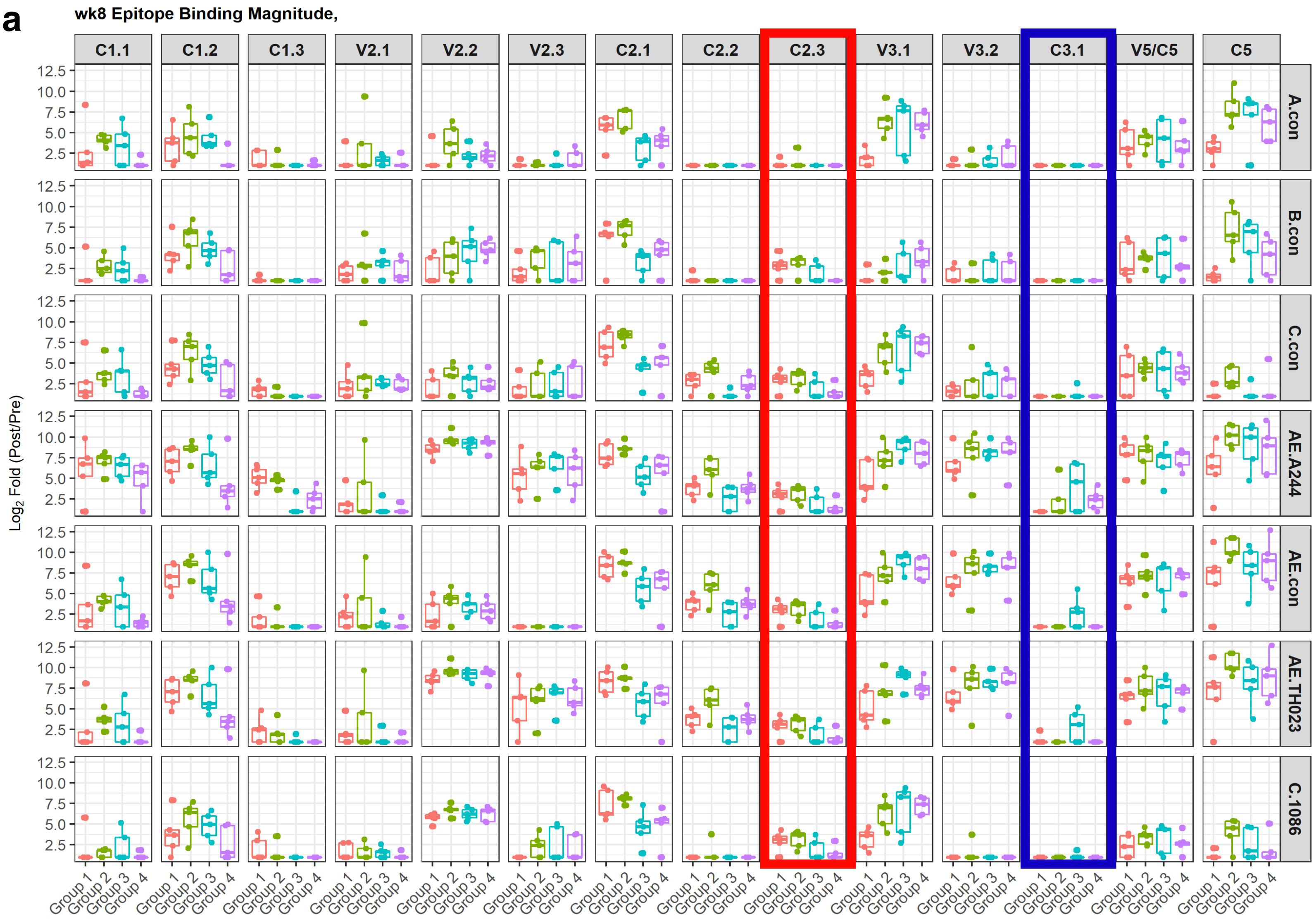

b

Epitope	Start Pep#	End Pep#	HXB2_Nbr
**C1.1**	24	26	aa71-77
**C1.2**	34	36	aa101-107
**C1.3**	39	41	aa116-122
**V2.1**	50	51	aa154-157
**V2.2**	53	54	aa163-166
**V2.3**	56	57	aa172-175
**C2.1**	66	67	aa202-205
**C2.2**	70	71	aa214-217
**C2.3**	84	86	aa256-262
**V3.1**	100	101	aa304-307
**V3.2**	103	105	aa315-321
**C3.1**	115	117	aa350-358
**V5/C5**	149	153	aa463-475
**C5**	156	158	aa484-490

A244 Δ11 gp120/Rehydragel
A244 Δ11 gp120/ALFQ
A244 Δ11 gp120 mRNA-LNP
A244.CD4KO Δ11 gp120 mRNA-LNP

Supplementary Figure 2. Clade-specific peptide binding antibody responses post mRNA-LNP and adjuvanted recombinant protein vaccination. a. Box-and-whisker plots of antibody binding magnitude for various Envs from different clades**.** The virus name is shown on the right. Env region is shown on the top of each column. Rectangles indicate differences in C2 (red) and C3 (blue) binding antibodies between mRNA and protein immunization. Env regions encompassed by the peptides are listed at the top of the column. **b.** Peptide locations within reference HXB2 envelope amino acid sequence.

Supplementary Figure 3

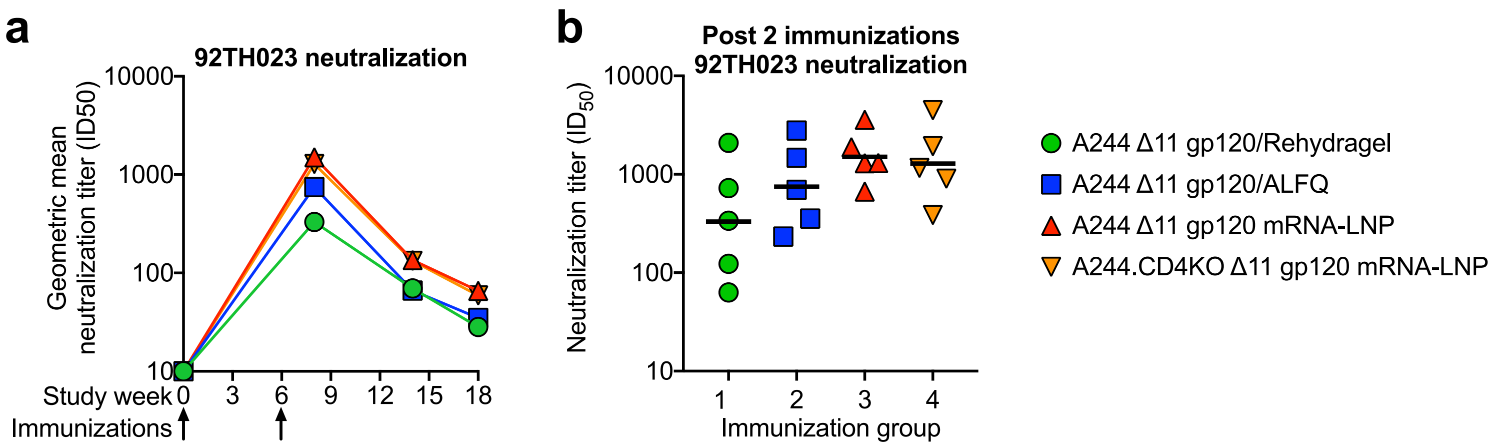

Supplementary Figure 3. Adjuvanted recombinant gp120 and mRNA-LNP-encoded gp120 immunizations elicit comparable HIV-1 serum HIV-1.92TH023 neutralizing antibodies. a. Serum neutralization of HIV-1.92TH023.6 infection of TZM-bl cells over the course of vaccination. The symbol represents group geometric mean. **b.** Comparison of neutralization ID50 titers two weeks post the second immunization. Neutralization titer is shown as ID50 reciprocal serum dilution. Horizontal bar represents group geometric mean.

Supplementary Table 1

Supplementary Table 1. Rhesus serum HIV-1 neutralization ID50 titers after 6-valent sequential SOSIP gp140 mRNA-LNP immunization.

		Monkey			
[1]Week	Virus	150252	150794	150796	150798
18	CH0505.w4.3	482	146	210	330
18	CH0505 TF	10	10	10	10
18	CH0505s/293S/GnTI-	10	10	10	10
18	CH0505TF.gly4	10	10	10	10
18	CH0505s.G458Y.4	10	10	10	10
18	CH0505s.G458Y.4/293S/GnTI-	10	10	10	10
32	CH0505s.G458Y.N279K.2/293S/GnTI-	10	48	32	10
32	CH0505TF.N279K/293S/GnTI- (M5)	10	10	10	10
18	TRO.11	10	10	10	10
18	[2]SVA-MLV	10	10	10	10

[1]Week 18 and 32 are two and 16 weeks after the fifth and final immunization respectively. [2]SVA-MLV, murine leukemia virus.

Supplementary Table 2

Supplementary Table 2. Rhesus serum HIV-1 neutralization ID50 titers 6-valent sequential gp160 mRNA-LNP immunization.

		Monkey			
[1]Week	Virus	150250	150793	150795	6858
18	CH0505.w4.3	1164	3111	4169	2495
18	CH0505 TF	10	10	10	10
18	001428-2.42	10	10	10	10
18	426c.N276D.N460D.N463D/293S/GnTI-	10	43	10	10
18	426c.N460D.N463D/293S/GnTI-	10	10	10	10
18	CH0505.w24.e12 (CH505.30.12)	10	10	10	10
18	CH0505.w24.e20 (CH505.30.20)	10	10	10	10
18	CH0505TF.M11 (CH505.M11)	10	10	10	10
18	CH0505TF.M5 (CH0505.M5)	10	10	10	10
18	CH0505TF.gly3.276	10	10	NT	10
18	HIV CH0505.C.e.B18 (CH505.136.B18)	10	10	10	10
18	HIV CH0505TF.gly4	10	10	10	10
18	[2]SVA-MLV	10	10	10	10

[1]Week 18 is two weeks after the fifth and final immunization. NT, not tested due to sample availability. [2]SVA-MLV, murine leukemia virus.

Supplementary Table 3

Supplementary Table 3. Rhesus serum HIV-1 neutralization ID50 titers after 6-valent sequential SOSIP gp140 protein/GLA-SE immunization.

		Monkey			
[1]Week	Virus	150251	6857	T244	T245
18	CH0505.w4.3	450	122	783	200
18	CH0505 TF	10	10	10	10
32	CH0505TF.N279K/293S/GnTI- (M5)	10	10	10	10
18	CH0505TF.gly4	10	62	10	10
18	CH0505s.G458Y.4	10	10	10	10
18	CH0505s.G458Y.4/293S/GnTI-	37	10	10	10
32	CH0505s.G458Y.N279K.2/293S/GnTI-	[3]NT	NT	10	24
18	CH0505s/293S/GnTI-	46	10	32	10
18	TRO.11	10	10	10	10
18	[2]SVA-MLV	10	10	10	10

Week 18 and 32 are two and 16 weeks after the fifth and final immunization respectively. [2]SVA-MLV, murine leukemia virus. [3]NT, not tested due to sample availability.
